# Editorial: The Versatile Role of Nicotinamide Adenine Dinucleotide in Immunity

**DOI:** 10.3389/fimmu.2021.810280

**Published:** 2021-11-29

**Authors:** Björn Rissiek, Andreas H. Guse, Sahil Adriouch, Santina Bruzzone

**Affiliations:** ^1^ Department of Neurology, University Medical Centre Hamburg-Eppendorf, Hamburg, Germany; ^2^ The Calcium Signaling Group, Department of Biochemistry and Molecular Cell Biology, University Medical Centre Hamburg-Eppendorf, Hamburg, Germany; ^3^ Normandie University, UNIROUEN, INSERM U1234, Pathophysiology, Autoimmunity, Neuromuscular Diseases and Regenerative THERapies (PANTHER), Rouen, France; ^4^ Department of Experimental Medicine, University of Genova, Genova, Italy

**Keywords:** NAD, CD38, ARTC2, ADP-ribosyl transferase, NAADP, AAV (Adeno-associated virus), nanobodies, immunity

Nicotinamide adenine dinucleotide (NAD) is a highly abundant intracellular molecule, well known for its role as energy currency. However, intracellularly, NAD and its metabolic products ADP-ribose (ADPR), 2’-deoxy-ADPR (from 2’deoxy-NAD), cyclic ADPR (cADPR) and nicotinic acid adenine dinucleotide phosphate (NAADP) are also important second messengers essential for leukocyte Ca^2+^ signaling (1). Metabolism of these signaling molecules, as well as their exact molecular targets, are still unknown in many aspects, despite their importance in the regulation of innate and adaptive immunity.

Outside of cells, only a few cell surface receptors were identified, i.e. P2Y1 and P2Y11, that can sense extracellular NAD. However, the signaling functions of NAD is broadened by various nucleotide-degrading ecto-enzymes that can generate a variety of other nucleotide metabolites: ecto-CD38 metabolizes NAD into ADPR and cADPR, ecto-nucleotide pyrophosphatases (ENPPs) such as ENPP1 generate adenosine monophosphate (AMP) directly from NAD or from ADPR, thereby providing substrate for CD73-catalyzed adenosine generation and ligands for P1 receptors. Ecto-ADP-ribosyltransferases (ARTCs) utilize extracellular NAD to covalently attach ADPR groups to arginine residues on different cell surface proteins, including P2X7, resulting in a post-translational modification that can significantly affect the function of the modified target (2). In summary, NAD and its metabolites inside and outside of cells can have a remarkable impact on many different regulatory pathways of immunity.

This Research topic comprises 9 articles containing six original research articles, two reviews and one perspective article, all focusing on the role and function of extracellular NAD and intracellular NAD-derived metabolites as modulators of immunity. Three original articles investigated the cell surface ADP-ribosyltransferase ARTC2.2 which utilizes NAD to ADP-ribosylate mainly arginine residues on various cell surface target proteins. Leutert et al. identified several new ARTC2.2. targets on murine T cells. After isolating proteins from T cells incubated with NAD and subsequent mass spectrometry analyses, they identified 93 ADP-ribosylated peptides corresponding to 67 distinct protein. The ecto-5′-nucleotidase CD73 was among the newly identified ARTC2.2 targets and Leutert et al. could demonstrate that ADP-ribosylation of CD73 diminishes its capability to convert adenosine monophosphate into adenosine. Of note, ARTC2.2 also exists in a soluble form, as it can be proteolytically cleaved from the cell surface (3). Based on that Menzel et al. investigated whether cytokines are potential targets for ADP-ribosylation by soluble ARTC2.2. They could demonstrate that several cytokines, such as interferon gamma (IFNγ), interleukin 17A (IL-17A), IL-2 and IL-6 can be ADP-ribosylated by soluble ARTC2.2. For IFNγ Menzel et al. identified the ADP-ribosylation site and revealed that the attachment of the ADP-ribose group diminished IFNγ-induced STAT1 phosphorylation in macrophages. Finally, Gondé et al. presented a methodological approach relying on adeno-associated viral vectors (AAVs) coding for nanobody-based biologics targeting ARTC2.2 or P2X7, a prominent target of ARTC2.2. Following intramuscular injection of AAV coding for ARTC2.2/P2X7 nanobodies, functional modulation of their respective target could be demonstrated *in vivo*. This approach was additionally used to express nanobody-based heavy-chain antibodies, eliciting long-term depletion of T cells expressing high levels of ARTC2.2 or P2X7. This approach extends the available tool box to study ARTC2.2 or P2X7 functions *in vivo* and offers the possibility to modulate the function of ARTC2.2 and/or P2X7, or to deplete cells expressing these targets.

CD38 is another ecto-enzyme utilizing NAD as substrate. It can serve as NAD glycohydrolase or as cyclic ADP-ribose synthase, generating ADP-ribose or cyclic ADP-ribose from NAD, respectively. With their review article, Morandi et al. provide an update on the role of CD38 in the anti-tumor immune response. They highlight the importance of pathways such as the CD38/CD203a-concerted generation of immunosuppressive adenosine from ADP-ribose, the impact of extracellular NAD level on the T cell mediated anti-tumor response and discuss the use of new pharmacological inhibitors of CD38. Along this line, Baum et al. describe in their original article the generation of several new CD38-targeting heavy-chain antibodies. Some of these biologics inhibited CD38 cyclase activity, measured by the inhibition of the conversion of nicotinamide guanine dinucleotide (NGD, an NAD analog) to cyclic guanosine diphosphate ribose (cGDPR). Further, some clones exhibited potent complement-dependent cytotoxicity against CD38-expressing tumor cell lines. Additionally to its role in the anti-tumor immune response, or as target in tumor immunotherapy, CD38 has been attributed both positive and negative modulatory role in autoimmunity, depending on the disease setting (4). In their original article, Martínez-Blanco et al. demonstrate that CD38-deficiency ameliorates disease symptoms in a graft-*versus*-host mouse model for systemic lupus erythematosus (SLE). In their model, *Cd38^–/–^* mice had lower frequencies of follicular helper T cells, germinal center B cells, plasma cells and T-bet^+^CD11c^hi^ B cells, and lower levels of anti-ssDNA autoantibodies when compared to WT B6 mice. With this knowledge, new therapeutic strategies based on e.g. CD38-blocking antibodies or pharmacological approaches targeting CD38 enzymatic activity may arise as promising potential option to treat SLE. Furthermore, CD38 is part of a network of intracellular and extracellular enzymes that regulate the synthesis or degradation of NAD. This network is completed by NAD-engaged receptors and NAD-derived metabolites and referred to as NADome. In their review article Audrito et al. highlight the role of the extracellular part of the NADome in immunomodulation and its possible implications for therapeutic approaches.

Inside the cells, NAD is the basis for the production of intracellular second messengers. NAADP, a potent trigger of Ca^2+^ release from intracellular stores (5), can be generated intracellularly in acidic lysosomes in a base-exchange reaction from nicotinamide adenine dinucleotide phosphate (NADP) and nicotinic acid. With their perspective article, Walseth and Guse provide a view back to the discovery of NAADP and forth to the identification of new NAADP receptors, such as HN1L/JPT2. Upon NAADP-induced Ca^2+^ release, Ca^2+^-microdomain formation occurs at the junctions between the plasma membrane (PM) and the endoplasmic reticulum (ER) in less than a second. In their original article, Gil et al. provide a computational model to describe the evolution of cytosolic and ER Ca^2+^ concentrations in a three-dimensional ER-PM junction.

In summary, the articles published within this Research Topic greatly contribute to further extending our knowledge on NAD and its metabolites as regulators of immunity, both inside and outside of cells.

## Author Contributions

All authors listed have contribution to this editorial and approved it for publication.

## Funding

This work was funded by the Deutsche Forschungsgemeinschaft (DFG) (project number 335447717; SFB1328, project A01 to AG) and by a grant from the Agence Nationale de la Recherche (ANR) to SA (ANR-18-CE92-0046).

## Conflict of Interest

The authors declare that the research was conducted in the absence of any commercial or financial relationships that could be construed as a potential conflict of interest.

## Publisher’s Note

All claims expressed in this article are solely those of the authors and do not necessarily represent those of their affiliated organizations, or those of the publisher, the editors and the reviewers. Any product that may be evaluated in this article, or claim that may be made by its manufacturer, is not guaranteed or endorsed by the publisher.
